# Quittor*Read before the Massachusetts Veterinary Medical Association.

**Published:** 1894-01

**Authors:** Williamson Bryden


### QUITTOR.*

By Williamson Bryden, V.S.

The *Veterinary Journal* for September, 1893, contains the last of a synopsis of Prof. MacQueen's article on Quittor, read by him before the Midland Counties Veterinary Medical Association, England. Its completion in the *Journal* was awaited with interest and expectation one waits for what a popular teacher has to say when the subject reviewed is ill understood by the profession, and often difficult to treat satisfactorily in practice.

From ten to twelve years ago I engaged in a controversy with members of the teaching and hospital staffs of the "American" Veterinary College, New York, on the subject of Quittor, but dropped the matter as my contention did not appear to be understood. I beg to submit, therefore, that I am somewhat disappointed to find Prof. MacQueen still practically taking the side I then opposed (Vide *American Veterinary Review*, page 373, November, 1881; vide *American Veterinary Review*, page 479, January, 1882; vide *American Veterinary Review*, page 531, February, 1882; vide *American Veterinary Review*, pages 572, 573, 575, 577, March, 1882). In this he was not only sustained, but endorsed and applauded by all present at the meeting where his article was read and discussed.

Judging from my own practical experiences with True Quittor, I beg your permission, and also Prof. MacQueen's, to state that I regret the fact that the confusing of Quittor and a number of other diseases, thought to resemble it, has been most unfortunate, some of them being the result of accidental and other injuries, the ætiology and therapeutics of which are entirely different from one another. This confusion seems largely to prevail in Europe as well as in America. Pathology regarded in this general way loses its significance, and never can lead to anything but confusion of the student and illogical treatment of the patient; never! to that professional progress demanded by earnest friends of the veterinary surgeon who dare *to think* for themselves. From the evidence before us, veterinary practice in this respect in 1893 is little if any in advance of the days of Markham and Solleysel.

* Read before the Massachusetts Veterinary Medical Association.

Prof. Gamgee, in his "Diseases of our Domestic Animals," seems to me to have had a truer conception of the ætiology of Quittor than any of the veterinary authors I have chanced to read so far.

In presuming to take the positive stand I do, with reference to what I have (rightly or wrongly) called the physiopathological class of diseases of the horse's feet, legs and other parts, I beg to explain, especially to practitioners like myself, who have only received the ordinary veterinary education of some twenty-five years ago, that although I may not describe the many interesting phenomena and *adverse tissue changes* taking place within a part like the hoof, in the limb, and *to* the "horny box" itself, with the exactness of a trained pathologist, I *can* state in all candor, however, that my observations are the result of actual experiences in practice, one of more than ordinary proportions, during a period of over twenty years, and embracing never less than from 500 to 1,000 street railroad horses, in addition to a mixed practice among all kinds of horses and cattle larger than the average number. Pardon my making the foregoing remarks about *my practice* and also for suggesting that one of the most important features of any diseased condition to the student is to have a proper appreciation of its ætiology (its history) from the first adverse tissue change. When the hoof, from unfavorable conditions, begins to change (all or in part) in form, size, or quality. and to crowd and encroach on the sub-horny, sub-coronary and other structures that secrete and nourish healthy tissues, or that renew and repair such as have become degraded and imperfect, even to those that are the deepest seated, lining the nutrient foramen, etc.; so deep seated, indeed, that no operation that is safe and practicable could possibly reach, or that nature could renew or repair, in less than the average time which she demands to remove such defective tissues and reproduce or transform into new. These adverse changes (degenerations) having been going on for perhaps a year or more, and having involved the whole quarter to its innermost recesses, she (nature) finally rebels, and a swelling with lameness and some discharge appears at the quarter coronet. This is True Quittor. In my experience *such a sinus could no more be cured in a month*, or even hastened, than an egg could be hatched by some patent incubator in one-eighth, one-quarter, or one-half of the time it could be done by an old hen. Such a Quittor could no more be *cured in a month* than a case of seedy-toe that had undermined the wall to within an inch of the coronet could be *renewed in a month.* These are prerogatives of Old Nature, that she never tolerates being trifled with or coaxed from her control, not even by a *bluff* operation. I contend, therefore, that what is claimed to be excision of the cartilage has no more to do with the *cure* of Quittor than punctured wounds of the foot or treads on the quarter have in *causing* Quittor. I mean true Quittor which *cannot be cured by excision until after* Tom, Dick and Harry have treated it for *over six* (*6*) *months.* Then some operator pretends to cut it out, charges a large fee, and it gets well in a month or so. He also gets the credit for a cure that he has no right to. The fact of the matter is, it has then, as it were, "run out," and would have got well in common course in spite of their interference. It could not then be prevented from getting well unless complicated by some unusual interference, sometimes called surgery, heroic surgery, *a very bastard surgery*, too, I consider such work.

The other cases that get well after an operation are those said to be caused by *treads and accidents*, which could not possibly cause True Quittor, not even by a coincidence. How could it? The early history of the case is entirely inconsistent and incompatible; the adverse changes in the tissues of the quarter have not prepared it for this form of disease.

Let us take for example a case where from a defective coronet the coffin bone has become adversely affected, the cartilage elements of the pedal articulation being most involved, the process or function of "selection and appropriation" has not been properly performed, and in the disturbed state of the parts, this cartilage element has been *transplanted or transferred* by the circulation to the wings of the os pedis; such a piece of cartilage might be pared off with impunity, but this is uncalled for patchwork, and quite another affair from a case where the quarter is all degenerated, a change that has been going on perhaps for months or years.

During the early adverse tissue changes of true quittor, the disease can be arrested, but not after the *sinus has opened in the usual way;* then an average period of over seven and a half months is demanded according to my experiences and philosophy; then, also, a cure can be guaranteed, and so can the time required to effect it be predicted with almost unvarying exactness. These are facts that can be demonstrated in every case.

What will the pathologist of the future think of the Veterinarians who flourished in 1893, and who could not discriminate between fistulæ, sinuses of the *foot*, the different sinuses of the *withers*, or the different sinuses of the *poll?* Their different ætiologies have not been considered separately, but have all been, in each case, mixed together and an average struck. Of what use are such averages? It never seems to have occurred to practitioners that cases like these, or that cases like so called *navicular disease*, and cases of *disease of the navicular bone* were entirely different things. In August, 1892, I was called to a finely bred mare due to foal last Spring; she had a faulty gait—she was a "paddler"—but carried her master some three miles to the station once or twice daily till November. She developed in August a condition of the withers which indicated fistula, which it finally proved to be. I ordered it not to be opened until "fly time" was past. A flaxseed poultice, made with five per cent. solution of carbolic acid was put in a thin bag and attached under the front of an old thin blanket every night; an abscess like a swelling soon organized, which I opened by drawing tapes through it in November. In a short time it was entirely cured. Her foal came along in due course, and she was again ready for *service by the horse*—a very vigorous young one.

Immediately after I was again notified that the *mare* had another fistula. Instead of a true fistula which it resembled, however, it proved to be the result of an accident, the stallion having bitten the withers when serving her. This recovered in about three or four weeks, from the fact that it was merely a simple wound to tissues that were healthy when bitten. Then again, we have all seen cases where, from the fact that the feet having become tender, and the muscles, ligaments, and other tissues of the shoulders, having become gradually changed from sympathy or otherwise the dorsal spines become injured when lying or rolling against the wall or floor, from repeated bruises or other causes, tardy healing sores are the result.

Almost a similar class of cases is found in connection with poll evil, which could not all have come from a simple blow, as formerly taught.

Sincerely hoping that my remarks will offend no one, for they are only intended to stimulate investigation in newer lines; to train the eyes that they may see differently and more accurately; to cultivate our powers of observation, that we may be better able to interpret Nature's laws in health and disease, and be able to discriminate between the different tissue changes, and know the history of such changes from the very beginning—then can we hope to logically predict the result of treatment, and the time required to accomplish cure or repair most successfully.

